# Urban Living Labs: how to enable inclusive transdisciplinary research?

**DOI:** 10.1186/s42854-021-00026-0

**Published:** 2021-11-17

**Authors:** Pia Laborgne, Epongue Ekille, Jochen Wendel, Andrea Pierce, Monika Heyder, Joanna Suchomska, Iulian Nichersu, Dragos Balaican, Krzysztof Ślebioda, Michał Wróblewski, Wojciech Goszczynski

**Affiliations:** 1 European Institute for Energy Research, Karlsruhe, Germany; 2 Yale University, New Haven, USA; 3 University of Delaware, Newark, USA; 4 Sustainable Development Laboratory, Torún, Poland; 5 Nicolaus Copernicus University, Torún, Poland; 6 Danube Delta National Institute, Tulcea, Romania

**Keywords:** Urban Living labs, Inclusiveness, Transdisciplinary research, Knowledge co-creation

## Abstract

The Urban Living Lab (ULL) approach has the potential to create enabling environments for social learning and to be a successful arena for innovative local collaboration in knowledge co-creation and experimentation in the context of research and practice in sustainability transitions. Nevertheless, complex issues such as the urban Food-Water-Energy (FWE) Nexus present a challenge to the realization of such ULL, especially regarding their inclusiveness.

We present ULL as a frame for a local knowledge co-creation and participation approach based on the project "Creating Interfaces - Building capacity for integrated governance at the Food-Water-Energy-nexus in cities on the water". This project aims at making FWE Nexus linkages better understandable to the stakeholders (citizens and associations, city government, science, businesses), and to facilitate cooperation and knowledge exchange among them. This paper focuses on and discusses inclusiveness as a key aspect and challenge of ULLs and on what literature and our experiences in this regard suggest for the advancement of the concept of ULL towards ULL 2.0. These findings often also relate to framing transdisciplinary research in a wider sense.

## Policy and practice recommendations

1. Inclusive and meaningful ULLs need time. Efforts of network building, reaching out to different groups, learning about different perspectives, needs, and interests require substantial efforts that tend to be easily underestimated.

2. ULL activities should build on and respond to local needs; therefore, local stakeholders should be involved in the whole process including project definition.

3. Research programmes and projects should be constructed in a co-creative way and enable or even enforce the active commitment of local stakeholders.

4. For the research process to engage and influence local policy, it is necessary to build a sense of ownership among local decision-makers and to shape a vision for change concerning the local system from the beginning of the process.

## Science highlights

1. Sustainability transitions of urban systems need enabling space for learning and experimentation. Such spaces can be created in the form of ULL.
2. Questions of inclusion and exclusion are closely tied to power relations. Linked to these, people are differently enabled to act and take part and social differences in power may be reproduced in ULL.
3. There is a need for further reflection on and definition of the different roles, sensibilities, and qualifications of the involved researchers and stakeholders in ULL regarding skills, co-ownership, power relations, and legitimacy as well as process-related aspects.

## Introduction and definition of the concept of Urban Living Labs

The complexity of urban systems demands sophisticated approaches that integrate knowledge and methods from different disciplines and academic, and non-academic experts as well as citizens. Sustainability transitions of urban systems need space for learning and experimentation. Such spaces can be created in the form of Urban Living Labs (ULL) as an experimental governance approach (Voytenko Palgan et al., 2016). ULL can foster innovative local cooperation in knowledge co-creation and experimentation in the research and practice to improve urban sustainability. We refer to two complementary strands of literature adopting either the term “transitions” or “transformations”. As Hölscher et al. (2018) explain in a systematic comparison, these two perspectives can “enrich each other” (Hölscher 2018: 1) as a duality, one more oriented towards society sub-systems, the other to large-scale societal changes. Wolfram (2016) conceptualizes a concept of “transformative capacity” (Wolfram 2016: 121) with a list of key components responding to the question of how cities are enabled to initiate and implement sustainable change. Open and inclusive participation, as well as related empowerment, represent a necessary condition for such urban transformative capacity (Wolfram et al. 2019). We claim that ULL can create a framework for initiating this but face challenges regarding their inclusiveness.

In this paper, ULLs are conceived as part of process-oriented forms of transformative science, based on a rich tradition of action research and embedded in transdisciplinary research (TDR). Following McCormick Hartmann, 2017: 4), “Urban Living Labs can be considered both as an arena (geographically or institutionally bounded spaces), and as an approach for intentional collaborative experimentation of researchers, citizens, companies and local governments.”The specific term “Urban Living Lab” was introduced by the Joint Programming Initiative Urban Europe (JPI UE) as a forum for innovation (Voytenko Palgan et al., 2016; JPI Urban Europe, 2013). ULLs are a specific form of Living Labs or Real World labs (e.g., Menny et al., 2018; Klautzer et al., 2020). We focus on the aspect and challenge of inclusiveness of ULL and on the question of how to enable the use of the inclusive ULL as a space for collaboration and joint knowledge production. The paper presents learnings from literature and experiences from three ULL that are part of the ongoing project "Creating Interfaces". This project explores the challenge of knowledge co-creation and participation surrounding the complex urban FWE Nexus.

There is a need for clarification and refining within the ULL concept and enabling frameworks. ULLs are widely referred to in literature but with different definitions and ways of realization. The essence remains often unclear (see e.g., Hossain et al., 2019).

McCormick Hartmann (2017) and Bulkeley et al. (2018) distinguish three ideal types of ULLs: strategic, civic, and grassroots (McCormick Hartmann, 2017) or strategic, civic, and organic (Bulkeley et al., 2018). The differences lie in the organization, change-orientation, and leadership of the labs (for an overview table see Bulkeley et al., 2018: 322). The focus of this paper (as in project Creating Interfaces) is on strategic ULLs linked to programmes such as JPI UE to develop and test approaches that build local capacity (Bulkeley et al., 2018). Such labs represent an attempt to bring transformative research into a specific transdisciplinary research format (Defila and Di Giulio, 2019; Beecroft et al., 2018, referring to the wider concept of “Real world laboratories”; see also GAIA special issue 2018). Ideally, labs pursue three goals: produce findings (research objectives), initiate transformation processes (practical objectives), and support learning processes (educational objectives) (for further insights see Beecroft et al., 2018). Based on the analysis of 22 examples, Voytenko Palgan et al. (2016) defined five characteristics of ULLs: geographical embeddedness, experimentation and learning, participation and user involvement, leadership and ownership, and evaluation and refinement.

Even though different notions and definitions of living labs exist (Voytenko Palgan et al., 2016; for an overview see e.g., Rogga et al., 2018), their transdisciplinary nature seems to be one core characteristic (Schäpke et al., 2017; Rogga et al., 2018). Thus, in the following, many claims we base on experiences in ULL relate to transdisciplinary research (TDR) and practice in general. The history of the concept of TDR dates much farther back than the living lab approach (Rogga et al., 2018 identify the roots in Kurt Lewin’s experimental action research 1946. The authors provide a systematic comparison of the two concepts). As Defila and Di Giulio (2019) postulate, a fundamental characteristic of TDR is the substantial taking part of practice actors in a project. ULLs are part of an alternative research paradigm (also referred to as Mode 2 science in literature, see e.g., Gibbons, 2000; Gustafsson, 2013), transforming the relations between research and practice as well as science and society and addressing the task of creating and maintaining collaborative spaces as a core activity of researchers focusing on real-world problems (Wittmayer and Schäpke, 2014). In this approach, researchers share the task of knowledge provision with other stakeholders and add the responsibility for the facilitation of societal exploration of sustainability pathways and negotiation processes (Ibid.). Besides observation and analysis, their role in this approach is to initiate and to catalyse (Hilger et al., 2018), thus “blurring” traditional boundaries (Wittmayer and Schäpke, 2014: 485; see also Hilger et al., 2018 for roles of researchers in Real World Labs and Pohl et al., 2010 for roles of researchers in knowledge co-production). This raises innumerable questions, for example regarding legitimacy, power, and normativity.

## Urban Living Labs and the Food-Water-Energy Nexus and the project "Creating Interfaces"

The urban Food-Water-Energy (FWE) metabolism and infrastructures present a complex socio-technical process of coevolution. Growing in importance in recent years, the FWE Nexus was first discussed at the World Economic Forum in 2008 (WEF - World Economic Forum, 2011) as a mechanism to promote the sustainable use of resources. It has evolved to incorporate various facets, additional components, and disciplinary perspectives. We claim that given the abstract nature of the FWE Nexus, research is lacking on the social perception of systems and systematic learning and synthesis regarding the translation of nexus research from research to practice and wider society as well as among different disciplines.

The project "Creating Interfaces - Building capacity for integrated governance at the Food-Water-Energy-nexus in cities on the water" addresses capacity building for the urban FWE Nexus, making the FWE linkages and interfaces understandable to the stakeholders that include city governments, researchers, local businesses, and citizens. The project is funded through the Sustainable Urbanization Global Initiative (SUGI) Nexus of JPI UE and the Belmont Forum and includes 10 partners from Europe and the United States (research institutions, NGO, SME). "Creating Interfaces" facilitates cooperation and knowledge exchange among involved stakeholders by developing and testing innovative approaches for local knowledge co-creation and participation through ULLs in three mid-size cities in Tulcea (Romania), Wilmington (USA), and Słupsk (Poland). The cities were chosen based on similar size, geographic position close to major water bodies, and similarities regarding social aspects, e.g., vulnerable populations. Additionally, existing relationships with project partners—providing access to local stakeholders and knowledge regarding local structures and institutions, topics, and needs—played a role.

The ULLs aim to initiate local knowledge co-creation and participation as well as integrative urban planning and urban sustainability. The activities strive at making visible the interlinkages of the urban FWE Nexus and to foster system integration in vision building, decision making, and planning. The approach is to create different kinds of interfaces between knowledge systems, stakeholders of the different systems, and citizen-science-policy/administration. These interfaces are achieved through digital tools (for data collection and communication), workshops, network building, and platforms including visualizations.

The project employs a transdisciplinary research design, including co-creation of digital tools, testing of communication methods, and addressing the inclusiveness of transdisciplinary research. Realizing inclusiveness is a major challenge for Urban Living Labs and the experiences in the project reveal key factors that can be the basis for further discussions on enabling and realizing sustainable transdisciplinary research.

## Inclusiveness of Urban Living Labs

As Díaz-Reviriego et al. (2019) point out, inclusiveness has both a procedural as well as a substantive dimension, thus related to the process and the outcomes. Inclusiveness does not necessarily mean achieving strict representativeness (see also Franz, 2015 and for a critical discussion on representativeness Bourdieu 1979). It is about considering the diversity of community members in their socio-economic conditions, cultural/ethnic backgrounds, age, gender, and profession, for example, and enabling broad participation. Related, researchers need to identify relevant publics and to anticipate persons overlooked and unorganized groups to avoid surprises later (Thomas, 1995).

Questions of inclusion and exclusion are closely tied to power relations. Power has material and symbolic dimensions and is based on three sorts of capital: social, cultural, and economical (for an overview and explanation see Bourdieu 1983). Depending on these, people are differently enabled to act and social differences are reproduced. This concept of power relations is useful for understanding the complexity and deeply rooted mechanisms of social distinction and exclusion. As Bourdieu points out in the example of a national poll on the French education system in 1969, the decision to express an opinion is in direct relation to the impression to have legitimization to do so (Bourdieu 1979). Bourdieu analyses how people internalize social differences into anticipation of social boundaries leading to self-attribution of a certain status and potentially to self-exclusion in societal fields and discourses (Ibid.). Citizens have different possibilities to engage with the urban transformation due to diverging levels of social, cultural, and economic capital that they have or rather have not acquired in their biographies and inherited from their families.

In a critical analysis of citizen participation in American community development programmes, Arnstein (1969) distinguished levels of participation in a “ladder of participation” (Arnstein, 1969: 2017) rating citizens’ power over decision-making. Without any power, participation risks being an “empty and frustrating process” (Arnstein, 1969: 216) with unequally distributed benefits. In the ULL context, it is thus paramount to transparently communicate where the included party will find itself on the ladder of participation (White, 1996; Heyder et al., 2021).

Additionally, participatory processes risk bringing more power to the powerful, with “entrenched and reproduced existing power relations” instead of challenging the “patterns of dominance” (White, 1996: 6). Fritz and Binder (2020) scrutinized different transdisciplinary participation projects relating them to power asymmetries. They analyzed the degree to which different involved practitioners (groups or individuals) influence: i) the problem definition, ii) the choice of knowledge production approach(es), and iii) the decision-making. Their research focused on actor groups, funding bodies, and practitioners. They found that, with (i) actor selection and (re-)positioning, (ii) agenda-setting, and (iii) rule-setting, “researchers primarily exert instrumental power over these three elements of participation, whereas practitioners as well as the funding body wield primarily structural and discursive power” (Fritz and Binder, 2020: 1). Agenda setting is particularly important since it is the set out for all further considerations, and the ULL will need to identify its agenda. In agenda-setting, power or power asymmetry is apparent. Agendas can be subject to elite control—which in the case of the ULL might be e.g., the project consortium or key project partners (incl. specific local stakeholders). The power to put a topic on the agenda convincing the parties that it is in everyone’s interest (first face of power) (Dahl, 1957), to limit the potential topics to those which are innocuous to one party (second face of power) (Barach & Baratz, 1962), omit information, creating information asymmetry, that allows for a narrative around those topics convenient to one party (third face of power) (Lukes, 2004). The latter hints at the aspect of knowledge in the concept of power and participation. All these might be difficult to observe, however, the implementers of a ULL need to be aware and transparent in how topics came to be important.

A key element of power is the access to information and the potential information asymmetries between participants. Information asymmetries might be reinforced by the shift from traditional community meetings to online formats, especially since the COVID 19 pandemic. The digital age promises increased access to information and with it the potential for transparent communication and decision making, as hallmarks of e-governance. However, as argued by different scholars (Albrecht, 2006; di Gennaro and Dutton, 2006; Willis and Tranter, 2006) this shift to enable all—regardless of time and location—might not occur throughout the population. The authors argue that it rather might support persons already engaged in political processes such as privileged groups in making their voice (better) heard. Digitalization may be cementing and possibly reinforcing the gap between active and passive citizens, or rather heard and unheard citizens (Margolis and Resnick, 2002; Norris, 2001, 2003), and therefore not changing or diminishing participatory inequalities (Bimber, 2003; Norris, 2003). In this context, participatory inequalities relate to variation in power and capacity to engage in political processes (Dahl 1971). The term is particularly salient today for online participation (Nielsen, 2006) and the concept of the “digital divide,” which denotes a disconnect of citizens who know “how” to participate online using technology from those who do not. Crucially, these digital inequalities depend on whether citizens have access to or the financial means to invest in internet connection and end devices (Van Dijk, 2006) and parallel income inequalities (Brady 2004). As Robinson et al. (2015) claim, digital inequality should be considered alongside more traditional forms of social inequalities. In the "Creating Interfaces" project, the Covid-19 pandemic forced some changes in the methods and dynamics of the ULL process. Both in Slupsk and Tulcea, workshops with citizens were organized online. However, they were attended by a limited number of people who were comfortable using online tools. This choice was to some extent exclusionary and less than ideal but allowed the research to proceed. In Wilmington, activities with citizens were put on hold due to concerns that an exclusively online process might threaten inclusiveness and legitimacy of the ULL.

Virtual or not, the involved actors need to leave their comfort zone (Defila and Di Giulio, 2019) and can face exposure (Ibid.) The ULL setting can provide a safe space that enables different stakeholders to experiment and initiate uncommon exchange and cooperation but the space needs adequate design to be inclusive.

Research that explicitly aims at transforming society, especially if publicly funded, has a specific commitment towards the public and regarding justification and accountability (Defila and Di Giulio, 2019). A key element is an orientation towards the “common good”, which includes integrating different societal groups (Ibid). When it comes to developing results that help the wider community, it is important to address all relevant groups to let them learn from each other (Ghodsvali et al., 2019). However, inclusiveness has not been adequately addressed so far and the involvement remains a practical and understudied challenge (Menny et al., 2018; Voytenko Palgan et al., 2016; Bulkeley et al., 2018). Additionally, participation does not guarantee that all participants can contribute in a meaningful way and take part in the outcomes (Díaz-Reviriego et al., 2019). This is especially a challenge regarding highly complex topics like the urban FWE Nexus.

Besides the above-mentioned aspect of common good orientation, ULLs need to capture real-life context and its complexities to produce meaningful knowledge for practice and research. To get the full contextual picture, inclusiveness must be addressed. We claim that bringing diverse stakeholders into the research process strengthens the outcomes of the research as it helps reveal the variety of issues, needs, and perspectives as well as ideas and solutions within a community.

In project "Creating Interfaces", the initial phase of research through document review and key informant interviews helped identify the relevant stakeholders to invite to participate in the ULL. This ensured that in all three cases we collected information about the main stakeholders at the local level who are somehow connected to the urban FWE nexus: public (local decision-makers, representatives of public institutions), social (NGO, associations, local public councils), scientific, business, and citizens. Stakeholders connected to some institutions, organisations, or entities were invited directly, citizens through project partners and an open invitation. The widest active stakeholder representation was achieved in the case of Tulcea. In all three cases, we involved stakeholders and representatives of authorities of each nexus component at the local level. The ones involved in the food and water component were more receptive than the ones responsible for the energy component. The food and water components are, to some extent, better represented at the local level and that respective local stakeholders have more local capacity and independence than the ones responsible for the energy component.

It proved most challenging to involve citizens in all three "Creating Interfaces" project cities, especially at the beginning of the ULL process. We tried to enable broad participation through public invitations and snowballed contacts, but we have not gotten the desired level or breadth of participation (in part due to the pandemic and especially in the case of Wilmington). Involving citizens was mostly achieved through going to where the target groups were: in Slupsk (as the target groups are parents), to kindergartens and a community centre; in Tulcea, contacting residents during visits to the study sites and inviting them to workshops; in Wilmington, cooperating with neighbourhood organizations and approaching citizens in community centres. This outreach process was interrupted by the pandemic.

## Key challenges and factors regarding inclusiveness of ULL

We claim that ULLs can be an effective approach to integrate local communities into research and experimentation in a meaningful and inclusive way, especially if diverse community groups are represented in the research process. However, there is a gap in research and literature on who is involved or excluded (Bulkeley et al., 2018) as well as specific guidelines and minimal precedent regarding inclusiveness, even though achieving inclusiveness presents an important practical challenge (Evans and Karvonen, 2013; Voytenko Palgan et al., 2016). Instead of expecting stakeholders to come to the researchers, projects need to take as many steps as possible to reach out to them, thus increasing the likelihood of the involved stakeholders accurately representing an area (Marvin et al., 2018) and different needs, knowledge, and perspectives.

Literature review, as well as exploratory interviews with project partners and an internal workshop within the "Creating Interfaces" project consortium, helped to identify key factors and challenges for achieving inclusiveness in ULLs.

### Time

Time plays an important role in ULL success and influences other important aspects of the ULL process such as building trust and networks. Simply put, it takes time to establish a good working knowledge of the respective context, specific stakeholders, as well as needs of different societal groups.

Time relates to the available time of project personnel and stakeholders, the planned length of the ULL project, and of the optimal procedures for involving stakeholders. Active and early involvement is important to ensure that stakeholders can shape the ULL process and for the creation of a common vision and the identification of needs (Menny et al., 2018). The level and way of involvement differ in the three typical stages of ULLs: 1. Design, 2. Implementation, 3. Evaluation (Menny et al., 2018). These stages may overlap in time (Ibid.). Due to the ongoing project work, the paper concentrates on the two first phases.

#### Design phase

In the project "Creating Interfaces", outreach was done by the local project partners. First, discussions took place during the proposal writing process (initial project design phase) with local stakeholders and a focus on the municipalities. This included an initial definition of themes and starting points.

#### Implementation phase

While the overall thematic focus is generally defined in the design phase (in dialogue with local stakeholders), during the implementation phase, all involved stakeholders should agree on needs, objectives, and methods (Beecroft et al., 2018) and ideally share a common mission and vision (Nevens et al., 2013). A challenge can be to find a common language and create a mutual understanding (Klautzer et al., 2020).

The agreement on needs, objectives, and methods was realized in the ULLs of Project "Creating Interfaces" through regular meetings and smaller workshops with stakeholders. Outreach to citizens started at the beginning of the project first by reaching out to involved institutions, community associations, press, and social media. In Tulcea, the local research partners identified as the main challenge for inclusiveness the comprehensive identification of local stakeholders that form and shape the urban FWE Nexus. This was mainly achieved by qualitative interviews and a survey. The study topic was already well known and researched in Tulcea, facilitating the process. In Wilmington and Słupsk, local partners faced difficulties in the design phase selecting a study topic given the abstract nature of the urban FWE Nexus concept (Wiegleb and Bruns, 2018).

In Słupsk, collaboration and identification of topics for further research and activities took place in close cooperation with the local government. Based on interviews with representatives of institutions, public entities, and non-governmental organizations, the project sought to uncover the aspect of the FWE Nexus that has the biggest potential for wide discussion, stakeholder engagement, and searching for solutions. The most promising starting point appeared to be food as well as water management. Further research through analysis of existing data and a survey conducted among Słupsk citizens (having children) showed that the topic of food in educational institutions was an important issue. The institution's previous experience, specifically public kindergartens, in improving the quality of nutrition helped engage the discussion. The research challenge, however, was to integrate the food discussion into the broader context of the FWE Nexus. Another issue was the choice of date and time for the public ULL workshop, which was held on a Saturday (a free day for many professions in Poland). This choice was meant as a way to meet the needs of the citizens, who were not obliged to leave their work to take part in the workshop. However, with a relatively low level of civic activity in Słupsk, citizens could be reluctant to dedicate half of a free day, especially regarding the specific target groups of parents. Similar challenges emerged in Wilmington as to the starting point topic and timing of the workshop.

An important challenge for all three ULL was the limited availability of the project team as well as of local stakeholders including citizens. Interviews with investigators in the project revealed an underestimation of the time required for the ULL implementation and capacity limitations due to the framework conditions of the project (e.g., funding).

### Trust and transparency

Even though trust and trust-building in ULLs are often mentioned as important aspects or even prerequisites of living labs, these aspects still lack thorough investigation (for analysis on trust in knowledge production on the example of biodiversity see Gustafsson, 2013). Beecroft et al. (2018) relate the importance of trust-building to the non-hierarchical and non-determined characteristics of living labs and to the need to build sustainable working relationships, adding that this requires enough time. Franz (2015) describes trust-building activities in living labs like sewing courses and small talk that bring people together and help to build a basis of trust for further activities. He also stresses that a shift of research strategies towards long-term engagement was necessary. Building trust takes time (Nevens et al., 2013) and evolves through relationship-building. Both trust and transparency require good reciprocal communication with the stakeholders before and during the ULL and towards the citizens. For this specifically, communication skills are needed that should ideally be fostered in the education of researchers and practitioners as well as inside transdisciplinary projects and programmes (Jaeger-Erben et al., 2018).

Transparency is an important basis for transdisciplinary cooperation (Daedlow et al., 2016). A lack of transparency regarding underlying interests and concerns as well as goals in a project and levels of involvement and influence could put the ULL research at risk of being illegitimate (Beecroft et al., 2018; Lux et al., 2019). Furthermore, different stakeholders bring in different expectations (Beecroft et al., 2018) and a lack of transparency can create false expectations and conflicts. Providing as much transparency as possible can reassure stakeholders about what they are participating in. It can prevent disappointment and may lead to more active and sustained involvement in the project. Obtaining a commitment from public officials supporting the effort can provide legitimacy and help to build trust among participants (Thomas, 1995). Further, scope, goals, and options must be clearly communicated.

In the "Creating Interfaces" ULL, relationship-building was started through exchange with stakeholders in the city administration and different institutions for exploring local needs and questions jointly addressing citizens in the next step. Because of the complex and abstract nature of the urban FWE nexus, efforts for explaining and linking this approach to local needs and problems were needed. Besides three major workshops similar in all ULL, the local partners kept in contact through meetings and smaller workshops. In all cases, addressing and involving the citizens was not easy and needed channels of personal contacts and contacts through institutions/associations close to them as additional ways of addressing them beyond classical media communication. Having a “foot in the door” locally (through local partners) was a key factor for success in this endeavour. In Słupsk, direct relations between kindergarten managers and parents helped to engage them in the activities.

It should be emphasized that the experience in Słupsk showed that the process of building trust may concern representatives of public institutions even more than the citizens themselves. In participatory processes, such as the ULL process, expectations and needs articulated by citizens are often treated as criticism of public institutions. The institutions are not always able to respond directly due to the procedures in force, staff resources, or other issues—such as resistance to change of thinking and behaviour resulting from long-lasting habits (such problems emerged in cooperation with kindergarten managers).

### Communication, translation, and learning

Targeted communication addressing different publics throughout the process and good communication skills present a key factor for inclusiveness and the success of the ULL. This is especially important for a complex topic such as the urban FWE Nexus, which most people do not immediately or easily understand and often needs explanation. Developing FWE Nexus literacies is a prerequisite for engaging stakeholders on meaningful solutions that suit the needs of the urban communities.

This factor touches on a fundamental challenge of transdisciplinary sustainability research: the need for translation. If the TDR approach is taken seriously, cooperation should go beyond noticing and considering separate disciplinary contributions towards some common understanding and also aim at producing mutual and transformational learning (Defila and Di Giulio, 2018; Mitchell et al., 2017).

In synthesizing knowledge for transdisciplinary FWE Nexus research, the SUGI and other nexus research projects have developed and applied different approaches for generating a comprehensive understanding of the urban FWE Nexus and translating it into “products” for use in science or by local stakeholders. Visualizations of the nexus provide a way for translating the concept and analysis between disciplines and to society, and a basis for understandable and relevant outputs of research. The different approaches, experiences, and lessons learned of the projects constitute a valuable potential for developing further sustainability and nexus research by cross-project knowledge transfer and synthesis. Stakeholders, on the other hand, can use visualizations to understand and communicate on such complex systems and system interlinkages, e.g., identifying constraints, interconnections, different perspectives, and unknowns (Bammer, 2019). Based on our experiences, we claim that this can enhance inclusiveness by enabling stakeholders to better understand the concept and interlinkages and thus for providing meaningful contributions in a knowledge co-creation process. The project "Creating Interfaces" experimented with ways of visualizing the urban FWE Nexus’ interconnection understandably and appealingly, in cooperation with local stakeholders and citizens. However, the impact of visualisation on learning and understanding cannot be reported yet due to the ongoing research.

The translation into visual content can also enhance mutual learning, one of the key characteristics of living labs identified in the literature (for insights in learning processes of ULL see e.g., Voytenko Palgan et al., 2016). However, “learning” also refers to a “learn and adapt” approach in the project responding to the need to integrate local knowledge and adapt the project according to local needs and expectations to make it meaningful for the local communities. This was undergone in all three ULL of the project "Creating Interfaces".

### Build on local needs, create value

To achieve active participation, stakeholders need an incentive to participate. Creating value by addressing local needs is one incentive for active stakeholder participation and for achieving a meaningful local impact (Beierle and Cayford, 2002). This incentive can be reinforced through commitments of local decision-makers to incorporate the co-created knowledge into practice (Thomas, 1995). In our ULLs, this proved to be a difficult aspect, especially in the lack of direct options for resources for doing so but also given dominant governance practices (e.g. silos).

Regarding the ULL process, user orientation in the sense of taking into account the potential users of the outcomes is one of the key aspects defined in the reflective framework for research in societal responsibility (Ferretti et al., 2016, Haller et al., 2016).

Working with local stakeholders, especially citizens, directly on the urban FWE Nexus proved difficult in all three ULL since the urban FWE concept proved to be too abstract and far from the participants' imminent concerns. In the "Creating Interfaces" project, the decision was taken to locally approach and tackle the FWE Nexus by entering through one specific nexus element (food, water, or energy). The respective element identified with stakeholders as the most relevant one during the project definition phase, allowed to base the local activities on expressed needs. The interlinkages were then explored, aiming to make the nexus more tangible. In Wilmington, a participatory modelling (PM) exercise on the local nexus interlinkages was realized with citizens and local stakeholders during the public ULL workshop. PM is defined “as a purposeful learning process for action that engages the implicit and explicit knowledge of stakeholders to create formalized and shared representations of reality” (Voinov et al., 2018). Including PM into the ULL workshops allows to create and understand interconnections of the urban FWE Nexus, to grasp needs, wishes, concerns, or possible conflicts as well as the interdependencies between the stakeholders [citation redacted].

In Słupsk, food in public institutions was chosen as an entry point, in Wilmington community energy, in Tulcea water, and specifically irrigation of local gardens. For the stakeholders involved in Słupsk, it was important to provide information about the health effects of food on children in kindergartens and about the origin of food. The issue of the relationship between food locality and quality was one of the important topics discussed during the ULL workshop in Słupsk. These expectations allowed us to link them to elements of the FWE Nexus (e.g., energy—transport of products, water consumption for food production, nutritional values, production conditions). Researchers can play a relevant role here in enhancing the visibility of non-obvious connections and interfaces between elements of the urban FWE Nexus. In Wilmington, the initial choice of linking the ULL to local community energy was changed to food waste reduction after the first ULL workshop and subsequent interviews. This topic proved to better meet the knowledge needs of the community as well as being more tangible.

### Network and ownership building as continuing process

Maintaining contact and providing ongoing feedback even while not engaging with stakeholders enables them to feel involved and valued throughout the project’s timescale, especially if it is long (Durham et al., 2014). Additionally, it is crucial to follow up with those involved after the project has run its course, to make sure that the research and information collected was beneficial in the long term for the community. How long the outcomes are sustained and have impact is strongly related to local ownership of the process and the outcomes and linked to the intensity of involvement (Wittmayer and Schäpke, 2014). Based on our experiences we claim that the nature of traditional research project funding does not facilitate this process of taking over (co-)ownership by local society. The importance of skills and resources for process facilitation and enabling of active involvement of local stakeholders is not adequately considered. The engagement, again, needs time and resources as well as local acceptance of the coordination of the ULL after the project ends.

## Discussion and conclusions

Experiences from tackling knowledge co-creation on the urban FWE Nexus show that inclusiveness, meaningfulness, and impact of ULLs demand specific skills for well taking up their new roles of facilitator and change agents (Hilger et al., 2018). A practical example regards good communication between science and society and translation between disciplines and between research and practice/citizens. A specific transdisciplinary qualification of researchers should thus be ensured and fostered. As Jaeger-Erben et al. (2018) conclude, transdisciplinary researchers face specific challenges and need capacity-building, e.g., regarding procedural problems related to different logics involved as well as for creating “appropriate interfaces around common aims and boundary objects” (Jaeger-Erben et al., 2018: 384). In their factsheet on transdisciplinarity (part of a reflective framework for research in social responsibility), Winkelmann et al. (2016) define the organization of transdisciplinary research as a continuous problem-oriented process of convergence and consideration for the integration of the perspectives of science and practice. This requires moderation, time, and openness to modify approaches and ways of thinking. Likewise, it includes negotiation of roles and tasks that evolve in the different stages of the project and transparency on who is involved, when, for what, and with which power to determine outcomes for avoiding frustration and (self-)exclusion effects. Articulating the different roles helps moderate expectations of the process and achieve co-ownership of the ULL with different stakeholders. Menny et al. (2018) rightly point out that it is less about maximizing participation but about the right form and time, also according to the issues at stake. The aim of creating inclusive ULL enhances the need of designing the process in an enabling way to allow all voices to be expressed and heard.

Our ULL experiences and literature offer insights for further elaboration of the approach towards ULL 2.0. To strengthen their transformative capacity, inclusive ULLs require:

- 1) **Rethinking and reframing roles and tasks of researchers, citizens, planners, policymakers, and other urban stakeholders**.
  There is a need for negotiation, co-ownership, co-created problem definitions, and a shared understanding of possible pathways. Thereby, the aspect of power needs to be reflected both as an enabling and disabling factor for meaningfully taking part and regarding degrees of power in the sense of influencing the outcomes of experimentation, knowledge co-creation, and finally planning and policymaking. This strongly includes the dimension of differing knowledge and (attributed) expertise which is especially relevant concerning a highly complex topic like the urban FWE Nexus. In all three ULL, the complexity of the FWE nexus presented a (perceived) barrier for local stakeholders with limited knowledge on the linkages of FWE to participate in debates on the future development of this nexus in their communities.
  Furthermore, flexibility regarding the process and the resources should be provided as well as ways of enabling additional stakeholders to take part and for defining specific tasks, roles and responsibilities during the process. This is particularly important in the context of knowledge-authority (power) relations, which can affect the shape of the process and the position of stakeholders, e.g., reducing the participation of non-expert citizens. Also, the position of stakeholders due to their expert knowledge (researchers, scientists) or position (local decision-makers) can have a symbolic influence potentially disrupting the process of co-creating knowledge. It also requires an awareness of these risks, especially on the part of the stakeholders responsible for facilitating the process.
  Active engagement needs time, skills, and resources. Being inclusive and open means to invite actively, to go to where the people are, and to create enabling environments considering different expectations and abilities. Inclusiveness also means that avenues used to contact and retain stakeholder involvement are as comprehensive as possible, making participation as accessible as possible. A closer look at different roles and different levels of inclusion (and exclusion), also regarding different moments in the ULL process, would further sharpen the concept and practice of ULLs (for existing classifications see e.g., Juujärvi and Pesso, 2013; Habibipour et al., 2020; Hilger et al., 2018). Same for the aspect of non-scientists as “competent knowledge producers” (Gustafsson, 2013: 43) and the integration of this co-produced knowledge into science, policy, and planning practice.
- 2) **Funding framework and rethinking research and science**
  Following Jaeger-Erben et al. (2018), bringing forward transformative TDR is not only about the individual capacities of involved researchers but also touches on the need for changing frameworks. Regarding future programmes, a way for redefining roles could be explicit co-ownership through demanding co-responsibility or (co-)coordination roles of non-scientific stakeholders. Further discussion is needed, e.g., for preventing bureaucratic barriers and respecting the freedom of science (including aims, goals, and methods).
  Currently, research funding is strongly based on the approach of science as taking up societal problems in the form of research questions and remaining in charge of the process and outcomes. These can then be used by society. In process-oriented research, the “messiness of the actual collaborative research process … can only be planned to a certain extent” (Wittmayer and Schäpke, 2014: 494f) and can typically include working for some time without tangible results (Nevens et al., 2013). Main impacts may be diffuse and difficult to measure, e.g., the creation of trust and empowerment. We agree with Schliwa et al. (2015) who postulate that the most difficult to measure impacts of living labs—changes in behaviour and norms—can have the greatest transformative potential. We would add to this the creation of enabling and empowering environments as key outcomes that can increase the inclusiveness of sustainability transition processes. Impact is enhanced through the wide inclusion of different social groups and stakeholders. Academic structures and evaluation criteria for research and researchers undervalue non-scientific outcomes and of fostering sustainability literacy in and empowerment of society (Jaeger-Erben et al., 2018). In sustainability research, the focus on societal impact and empowering civil society to transform their environment should remain without losing connection to science and scientific discourse. Rethinking academic qualification and professional pathways (see also Jaeger-Erben et al., 2018) would be important for strengthening TDR.
  Ideally, the research process should be designed actively including different stakeholders and there should be a strong involvement of non-scientific stakeholders in the whole process. There are even examples of actively involving urban stakeholders in the design of research programmes (e.g., by JPI Urban Europe for its research agenda). This is a good start. We claim that the possibilities for engaging local stakeholders including citizens throughout the whole process in an active and meaningful way could be strengthened through financial setting points and formal prerequisites.
- 3) The ULL examples of the project "Creating Interfaces" illustrate the importance of **basing living labs on local needs, getting access to local communities** through local stakeholders as well as the aspect of trust and trust-building. Based on this, we argue that ULL should be strongly embedded or even rooted in their local context and provide openness to integrate responding to the different needs and interests.
  As a basis for this, there is a strong need for exploring the context and actor constellations, including power relations, and for developing relationships and different ways of enabling people to take part. Therefore, ideally, there would be time and resources for exploring the field and for collectively negotiating the project, e.g., in the form of exploratory funding. The next generation of ULLs 2.0 should include deliberate efforts on behalf of the research team to do thorough research into the communities' culture and history in which they are going. This also helps bring the project to where stakeholders are. In our experience, one of the most important factors to create opportunities for participation is to identify the places where stakeholders mingle and meet for various community activities. It is also important to identify a time that is comfortable for stakeholders for additional (non-work) activities. Activities should take place where people are comfortable being, and at times of the day and year that work for them. This is crucial for drawing in all potential stakeholders (Durham et al., 2014), and for lowering their efforts. Another way to attract and identify relevant participants is using existing stakeholder networks and creating a bottom-up process, literally knocking on doors around the neighbourhoods and building trust (Durham et al., 2014; Marvin et al., 2018).
  Frames for ULL 2.0 should provide openness for co-creative ways of defining needs, challenges, and solutions. The focus should shift from ‘solutionism’ to learning and experimenting with a challenge-driven approach.
- 4) **The possibility of gaining additional resources based on outcomes of the process and for their exploitation** would be fruitful and enable engagement in and local ownership of the ULL. This could also help for enabling the sustaining and handing over of responsibility as well as for further experimentation and replication. Especially in the ULL Tulcea, city stakeholders expressed the concern that solutions derived in the ULL process may be considered expensive or hard to finance by involved public authorities. A possibility to finance selected outcomes would enhance motivation and also inclusiveness regarding the outcomes. E.g., in Tulcea ideas regarding sustainable irrigation of the gardens could have been further developed as pilot projects and concrete outcomes. In the case of Słupsk, on the other hand, the implementation of the ULL process outcomes may be difficult due to the procedures and the current system of functioning of the institution, which would require changes. This aspect also refers to a need for fostering mechanisms for realizing the transfer of responsibilities and appropriation of the activities by local stakeholders for sustaining and embedding outcomes and initiatives. It seems crucial to conduct the ULL process from the beginning in such a way, as to think not only about its scientific value but its usefulness to stakeholders and its potential for future use. For the research process to engage and influence local policy, it is important to build a sense of ownership among local decision-makers. It is necessary to shape a vision for change of the local system from the beginning of the process. It is also crucial to identify the owners of the process, which should also be an outcome of the process, not a decision at the end.
- 5) Finally, **ULL 2.0 should be strengthened regarding their sustainability and transformative capacity in a long-term perspective**. The project-based nature of classical research-based ULL can set impulses, experiment with and demonstrate new ways of organizing societal problem-solving, and facilitate learning. The question is how these impulses and their outcomes can be sustained after the project ends and the researchers/initiators leave? How can the outcomes be systematically linked to and transform policy and planning? Here again, a key aspect would be funding enabling or even enforcing the commitment of the respective stakeholders through planning or implementation grants, or technical assistance and kinds of “seed” or “take it further” funding for setting up long-term initiatives and structures. This funding would also enhance the legitimacy of strategic ULLs and help for gaining transparency for citizens regarding the impacts their engagement can have. Another path could be long-term labs providing space and basic resources for experimentation.

In conclusion, inclusiveness and meaningfulness to local society are key points regarding the legitimacy and impact of ULLs as well as for ensuring the value of the scientific outcomes. When rethinking the concept of ULLs towards ULL 2.0, these should be strengthened in a co-creative process.

## Data Availability

Conclusions are based on the provided references as well as on experiences in the project and two exhaustive exploratory qualitative open interviews inside the Creating project team as well as an internal self-reflection project workshop.

## Abbreviations

ULL: Urban Living Lab
FWE Nexus: Food-Water-Energy nexus
JPI UE: Joint Programming Initiative Urban Europe
PM: Participatory Modeling
SME: Small and Medium Size Enterprises
SUGI Nexus: Sustainable Urban Initiative Food Water Energy Nexus
TDR: Transdisciplinary Research
WEF: World Economic Forum
